# Bleeding in Italy

**Published:** 1861-09

**Authors:** 


BLEEDING IN ITALY.—A correspondent of the *Medical Times and Gazette*, speaking of the medical practice in Italy, states that the use of the lancet in that country is as general as the use of castor oil or tamarind water. Both the physicians and the people are agreed upon the necessity of bleeding, not only as an antiphlogistic remedy, but as a hygienic measure, as something good in itself. A practice very similar to this prevailed, not more than twenty-five years ago, in many parts of Pennsylvania, especially among the German population. People insisted upon being bled at least twice a year, that is, in the spring and autumn, believing that the operation exercised a depuratory influence upon the system. Now no one uses the lancet, either as a preventive or as a curative of disease. Brunonianism and Toddism are rampant.

